# Inhibition of nSMase2 Reduces the Transfer of Oligomeric α-Synuclein Irrespective of Hypoxia

**DOI:** 10.3389/fnmol.2019.00200

**Published:** 2019-08-28

**Authors:** Valerie Sackmann, Maitrayee Sardar Sinha, Christopher Sackmann, Livia Civitelli, Joakim Bergström, Anna Ansell-Schultz, Martin Hallbeck

**Affiliations:** ^1^Department of Clinical Pathology, Linköping University, Linköping, Sweden; ^2^Department of Clinical and Experimental Medicine, Linköping University, Linköping, Sweden; ^3^Department of Public Health and Caring Sciences, Uppsala University, Uppsala, Sweden

**Keywords:** Parkinson’s disease, extracellular vesicles, neutral sphingomyelinase 2, α-syn, hypoxia, cell-to-cell transmission, sphingomyelin, ceramide

## Abstract

Recently, extracellular vesicles (EVs), such as exosomes, have been proposed to play an influential role in the cell-to-cell spread of neurodegenerative diseases, including the intercellular transmission of α-synuclein (α-syn). However, the regulation of EV biogenesis and its relation to Parkinson’s disease (PD) is only partially understood. The generation of EVs through the ESCRT-independent pathway depends on the hydrolysis of sphingomyelin by neutral sphingomyelinase 2 (nSMase2) to produce ceramide, which causes the membrane of endosomal multivesicular bodies to bud inward. nSMase2 is sensitive to oxidative stress, a common process in PD brains; however, little is known about the role of sphingomyelin metabolism in the pathogenesis of PD. This is the first study to show that inhibiting nSMase2 decreases the transfer of oligomeric aggregates of α-syn between neuron-like cells. Furthermore, it reduced the accumulation and aggregation of high-molecular-weight α-syn. Hypoxia, as a model of oxidative stress, reduced the levels of nSMase2, but not its enzymatic activity, and significantly altered the lipid composition of cells without affecting EV abundance or the transfer of α-syn. These data show that altering sphingolipids can mitigate the spread of α-syn, even under hypoxic conditions, potentially suppressing PD progression.

## Introduction

Parkinson’s disease (PD) is a progressive age-related movement disorder that results in the selective loss of midbrain dopaminergic neurons and widespread pathology at later stages of the disease (Braak et al., 2003). PD is also characterized by intracellular deposits of aggregated α-synuclein (α-syn), known as Lewy bodies and Lewy neurites, and research indicates that soluble oligomeric α-syn (oα-syn) is the most toxic species [reviewed in Bengoa-Vergniory et al. (2017)]. Anatomical connections and cell-to-cell contact promote the accumulation of these aggregates. The mechanisms of this spreading, including the important role of cell-surface proteins (Reyes et al., 2019) and extracellular vesicles (EVs) (Russo et al., 2012), are beginning to be elucidated. Small EVs, specifically exosomes, are small membranous vesicles (30–150 nm) that are formed by the invagination and budding of endosomal multivesicular bodies from the membrane (Van Niel et al., 2006). These vesicles can serve as vehicles for transferring proteins, lipids, mRNA, and miRNA between cells. Intercellular communication involves the transfer of EVs and their functional biomolecular contents between cells and might be a prominent method by which oα-syn is transferred in PD.

The biogenesis of EVs depends on pathways that are either dependent or independent on endosomal sorting complexes required for transport (ESCRT) machinery [reviewed in Kowal et al. (2014)]. There are four neutral sphingomyelinase (nSMase) isoforms, nSMase1, nSMase2, nSMase3, and mitochondria-associated SMase, each encoded by the genes *SMPD 2–5*, respectively (Hofmann et al., 2000). However, nSMase2 is the most abundant sphingomyelinase isoform in the brain (Shamseddine et al., 2015). nSMase hydrolyzes sphingomyelin (SM), a type of sphingolipid, to produce ceramide (Cer), which forms EVs through the ESCRT-independent machinery pathway (Marsh and van Meer, 2008; Trajkovic et al., 2008). Although it was reported nearly half a century ago that SM accumulates in Lewy bodies (den Jager, 1969), little is known about how SM metabolism relates to the pathogenesis of PD. The importance of SM metabolism is supported by reports showing increased levels of Cer in the plasma of PD patients (Mielke et al., 2013; Xing et al., 2016; Savica et al., 2016). The regulation of Cer in the brains of PD patients seems more complex as both increased Cer levels in the visual cortex (Cheng et al., 2011) and decreased Cer levels in the anterior cingulate region (Abbott et al., 2014; Murphy et al., 2014) have been reported. The inhibition of nSMase2 has been shown to reduce the secretion of EVs [reviewed in Bienias et al. (2016)], but how nSMase2 influences PD pathogenesis in terms of protein accumulation and cell-to-cell transfer has not been studied so far.

Aging, which is associated with numerous changes in the brain, including alterations in enzymatic activities, lipid metabolism, and oxidative stress, is a major risk factor for neurodegenerative diseases such as PD (Anderton, 1997;Valli et al., 2015), and these alterations are known to induce α-syn aggregation (Hashimoto et al., 1999; Scudamore et al., 2018). Oxidative stress can also cause the post-translational modification (PTM) of α-syn, including modification by 4-hydroxy-2-non-enal (HNE), an end product of lipid peroxidation that promotes α-syn oligomerization, aggregation, and cytotoxicity (Klucken et al., 2013). Furthermore, HNE PTMs have recently been reported to increase the secretion of EVs containing cytotoxic oα-syn (Zhang et al., 2018), and lipid vesicles affect the aggregation of HNE-modified α-syn (Sardar Sinha et al., 2018). In addition, nSMase2 is sensitive to oxidative stress (Levy et al., 2006; Castillo et al., 2007) and has been shown to increase with age (Rutkute et al., 2007; Vozella et al., 2017).

This study investigated nSMase2 as a possible driving force for the progressive accumulation of α-syn and its transfer between neuron-like cells. Since α-syn PTMs can be induced by oxidative stress (Duce et al., 2017), we investigated the influence of hypoxia on the SM–Cer pathway with regard to promoting α-syn aggregation and transfer. For the first time, we showed that the inhibition of nSMase2 leads to a significant reduction in HNE-modified oα-syn aggregation and transfer between neuron-like cells. Hypoxia decreased nSMase2 protein levels but did not affect total EV abundance or alter the oα-syn transfer rate. These data provide evidence that SM metabolism may play a larger role in PD pathogenesis and support the need for additional investigations of sphingolipids in PD patients.

## Materials and Methods

### Preparation of HNE-oα-syn

For oligomerization, recombinant human α-syn (AlexoTech) was prepared as previously described (Näsström et al., 2011; Domert et al., 2016; Sardar Sinha et al., 2018). α-Syn was dissolved in 20 mM Tris-buffered saline (TBS) (pH 7.4), and 140 μM α-syn monomers were incubated with 2.8 mM HNE for 7 days at 37°C without agitation. After 7 days, the protein mixture was desalted by a Zeba desalt spin column (Thermo Scientific), equilibrated with Na_2_CO_3_ (0.1 M, pH 8.5) and incubated with the fluorophore Alexa Fluor 700 (AF700) succinimidyl ester (final concentration of 1.58 mM, Life Technologies) for 1 h at room temperature with shaking.

Labeled HNE-oα-syn was separated from free dye by size exclusion chromatography, which was also used to separate the labeled oligomers from the mixture. A Superose 6 PC 3.2/30 GL column (GE Healthcare) coupled to a liquid chromatography system (ÄKTA pure, GE Healthcare) was equilibrated with NH_4_HCO_3_ (50 mM, pH 8.5), and 500 μl of sample was injected into the column. To estimate the molecular weight of the α-syn species, LMW gel filtration calibration kits (GE Healthcare) were used. Oligomeric and monomeric α-syn species were eluted at a flow rate of 0.5 ml/min, collected, and lyophilized. The final pellet was resuspended in TBS buffer, and the protein concentration was determined using a Nanodrop (ND-1000 Spectrophotometer). The oα-syn used during the *in vitro* studies had a final concentration of 1 μM and an incubation period of 3 h in serum-free culture media.

### Transmission Electron Microscopy

For transmission electron microscopy (TEM) analysis, 5 μl of HNE-modified oα-syn was placed onto carbon-coated copper grids and incubated for 5–10 min. After removing the excess liquid, the grids were washed two times with deionized water prior to negative staining with 2% uranyl acetate for 30 s. The samples were analyzed with a JEM-1230-EX electron microscope (Jeol).

### Differentiation and 3D Coculture of SH-SY5Y Cells

**FIGURE 1:**
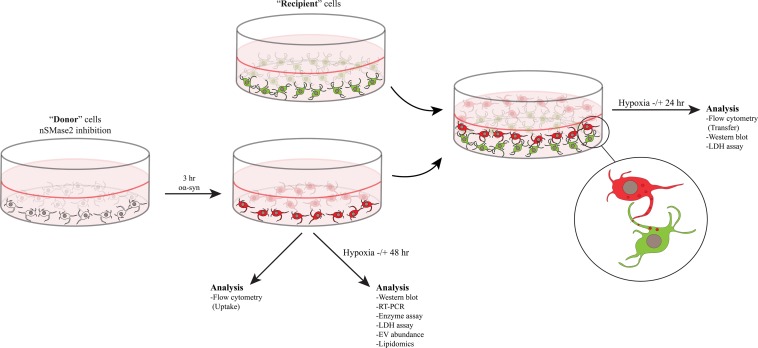
nSMase2 was inhibited in donor cells and then treated with oα-syn (labeled with AF700) for 3 h. The donor cells were then immediately analyzed for the uptake of oα-syn or were cultured in the presence or absence of hypoxic conditions for 48 h for further analysis. Coculture experiments were carried out by placing the donor cells with oα-syn on top of fully differentiated recipient cells stably expressing β-actin-GFP. Cocultured cells were incubated in the presence or absence of hypoxic conditions for 24 h before analysis.

Differentiation and coculture were performed as described previously, with minor modifications (Agholme et al., 2010; Nath et al., 2012; Domert et al., 2016; Sackmann et al., 2017). All SH-SY5Y cells were cultured in complete culture media containing MEM-GlutaMAX (Gibco) supplemented with 10% fetal bovine serum (PAA Laboratories), 50 U/ml penicillin, 50 μg/ml streptomycin, and 2 mM L-glutamine (all from Gibco) at 37°C with 5% CO_2_. Donor cells were differentiated with 10 μM retinoic acid (RA; Sigma Aldrich) for 7 days and seeded at a density of 1400 cells/cm^2^. nSMase2 was inhibited in donor cells, as described below, and the cells were then treated with oα-syn, where applicable. For the coculture experiments, SH-SY5Y cells that stably expressed β-actin-GFP (Addgene plasmid #21948, provided by Ryohei Yasuda) were used to distinguish the recipient cells from the donor cells. Recipient cells were prepared by seeding RA-differentiated GFP cells at a density of 1700 cells/cm^2^ and allowing them to further differentiate for 10 days in a 20% Extracellular Matrix (ECM) Gel coating (Corning) with a growth factor cocktail in serum-free culture media [50 ng/ml brain-derived neurotropic factor (PeproTech), 10 ng/ml neuregulin β1 (R&D Systems), 10 ng/ml nerve growth factor (R&D Systems), and 24 nM vitamin D_3_ (Sigma-Aldrich)]. Finally, coculture was carried out by trypsinizing the donor cells, placing them on top of the recipient cells, and incubating the coculture for 24 h at 37°C to allow the formation of synaptic-like connections. A graphical schematic of the experimental setup is presented in Figure 1.

### siRNA Transfection

Donor cells were seeded 24 h before transfection. Small interfering RNA (siRNA) transfection was performed by targeting nSMase2 (*SMPD3* gene) with siRNA or negative control (All Stars Negative Control siRNA) using HiPerFect Transfection Reagent (all from Qiagen), according to the manufacturer’s instructions. A siRNA concentration of 20 nmol/L in Opti-MEM + GlutaMAX (Gibco) media was used for transfection, and the transfection period was 6 h, after which the transfection media was removed and the cells were incubated in serum-free culture media for an additional 42 h before the addition of oα-syn, where applicable. The selected predesigned FlexiTube siRNA target sequence was *SMPD3*, 5′-CACGAACGGCCTGTACGATGA-3′. Knockdown was verified by immunoblotting and RT-PCR, and a downregulation of 70% was considered sufficient.

### CRISPR/Cas9 Cells

CRISPR/Cas9 targets were designed against the *SMPD3* gene (Ensembl ENSG00000103056) using the software available on Benchling (Benchling Inc.). A guide RNA (gRNA) targeting exon 3, the first coding exon, was constructed using the gRNA sequence 5′-CATGGTCGAAGGCCTCGTCG-3′, resulting in a truncated nSMase2 protein. The locus targeted by the gRNA occurs upstream of the nSMase2 catalytic domain, specifically residues D430 and K435, which are responsible for SM hydrolysis (Airola et al., 2017). CRISPR/Cas9 plasmid design and assembly were performed according to the instructions of Ran et al. (Ran et al., 2013) using the plasmid pSpCas9(BB)-2A-GFP (PX458) (Addgene plasmid #48138, provided by Feng Zhang). Wild-type (WT) SH-SY5Y cells were transfected and single-cell sorted, and clonal cell lines were screened for anticipated changes to nSMase2 protein expression by Western blot and immunocytochemistry. As predicted, the selected clone was undetectable by nSMase2 antibodies (Supplementary Figure S1). The selected nSMase2 knockout (KO) clone was used in subsequent studies for differentiating cells into donor cells, as described above.

### Cambinol Treatment

Cambinol [5-(2-hydroxynaphthalen-1-ylmethyl)-6-phenyl-2-thioxo-2,3-dihydro-1H-pyrimidin-4-one; Sigma-Aldrich], a pharmaceutical inhibitor of nSMase2 (Figuera-Losada et al., 2015), was prepared at a stock concentration of 20 mM in DMSO and added to donor cells at a final concentration of 10 μM for 48 h in serum-free culture media.

### Hypoxic Exposure

To produce oxidative stress, the cells were incubated in a hypoxic incubator set to 1% O_2_. For experiments analyzing donor cells alone, the cells were incubated for 48 h following siRNA transfection and/or oα-syn treatment. For coculture experiments, the cells were exposed to hypoxic conditions for 24 h after the establishment of the coculture.

### Western Blot

Cells were lysed with Tris-HCl (0.5 M, pH 6.8), 10% glycerol, and 2% SDS. For each sample, 20 μg of protein and 0.1 M DTT was loaded on a 4–20% SDS ClearPAGE gel. EVs (isolated as described below) were lysed in RIPA buffer [50 mM Tris-HCl, 150 mM NaCl, 1% Triton X-100, 0.5% sodium deoxycholate, 0.1% SDS, 40 μl/ml PhosSTOP (Roche) phosphatase inhibitor, and 10 μl/ml Halt protease inhibitor (Thermo Fisher)] followed by vigorous vortexing. EV lysates were mixed with 4 × Laemmli loading buffer and DTT and loaded onto a 4–20% SDS ClearPAGE gel. The proteins were transferred onto nitrocellulose membranes (Invitrogen). The blots were incubated with the following antibodies, as indicated: mouse anti-β-actin (1:10,000, A5441, Sigma-Aldrich), rabbit anti-nSMase2 (1:1,000, PA5-49140, Thermo Fisher), rabbit anti-α-syn (1:1,000, 701085, Invitrogen), mouse anti-pS129 (1:1000, 015-25191, Wako), and mouse anti-flotillin-1 (1:1,000, 610820, BD Transduction Laboratories). Goat anti-mouse HRP or goat anti-rabbit HRP (Dako) were used at a 1:2,000 dilution as a secondary antibody. The blots were visualized using Clarity ECL (BioRad) on a ChemiDoc MP imaging system (BioRad) and analyzed by ImageJ software.

### Semiquantitative RT-PCR

Total RNA was isolated using the RNeasy Mini Kit (Qiagen) and converted to cDNA using a High Capacity RNA to cDNA kit (ThermoFisher) following the manufacturer’s instructions. Semiquantitative real-time PCR (RT-PCR) was performed using the TaqMan Gene Expression Kit and with FAM/MGB probes [*SMPD3*: Hs00920354_m1; *SMPD2*: Hs00906924_g1; *SMPD1*: Hs04183448_m1; *TSG101*: Hs01121709_m1; HGS: Hs00610371_m1; *STAM*: Hs00989740_m1; *VPS4A*: Hs00203085_ m1; *CHMP4A*: Hs00204331_m1; ALIX (*PDCD6IP*): Hs0099 4346_m1; *FLOT2*: Hs01080468_g1; *PLD2*: Hs01093216_m1; *CD81*: Hs01002167_m1; *SNCA*: Hs01103383_m1; β-actin (*ACTB*): Hs01060665_g1; all from Applied Biosystems]. Reactions were carried out in triplicate using a 7500 Fast Real-Time PCR system (Applied BioSystems). The data were analyzed according to the comparative Ct method to determine the fold change in the expression levels relative to the control samples, and statistics were performed on the ΔΔC*_t_* values.

### Flow Cytometry

oα-syn uptake and transfer were quantified by flow cytometry. Cocultured cells were recovered from the ECM gel using Corning Recovery Solution (Corning) according to the manufacturer’s instructions, while donor cells used to determine the uptake of oα-syn were trypsinized for 1 min. The cells were then resuspended in PBS and filtered through CellTrics 30-μm filters (Sysmex), subsequently analyzed using a Beckman Coulter Gallios and analyzed with Kaluza 1.3 software. For transfer, forward and side scatter were gated to identify the cell population. WT and GFP-only controls were used to identify gating for AF700- and GFP-negative cells, and recipient cells with no transfer. Positive transfer was identified with double fluorescence. The ratio of transfer was normalized to the percentage of double fluorescent cells in the control coculture. The ratio of uptake was normalized to the percentage of AF700 cells, as this was performed in WT cells.

### Immunocytochemistry

Cells used for imaging were seeded on top of glass coverslips, fixed with 4% PFA in PBS, and permeabilized with incubation buffer (0.1% saponin and 5% FBS) for 20 min at room temperature. The cells were incubated with primary antibodies against C-terminal nSMase2 (PA5-49140, ThermoFisher) or N-terminal nSMase2 (SP4061, ECM BioSciences) at 1:500 in incubation buffer overnight at 4°C. This was followed by washing and incubation with Alexa Fluor 488 goat anti-rabbit (1:400; Life Technology) for 75 min at room temperature. After washing in PBS, the slides were mounted with ProLong containing DAPI (ThermoFisher). Images were acquired with a Zeiss LSM 700 inverted confocal microscope using a 20 × objective lens.

### LDH Toxicity Assay

Cytotoxicity was determined by the presence of lactate dehydrogenase (LDH) according to the manufacturer’s instructions (Pierce) and analyzed at 490 nm and 650 nm using a VersaMax ELISA plate reader.

### nSMase Enzyme Activity Assay

nSMase enzymatic activity was determined using the nSMase assay kit from Echelon Biosciences following the manufacturer’s instructions. Donor cells were harvested and washed with PBS; resuspended and lysed in 25 mM Tris–HCl, 150 mM NaCl, and 1% Triton X-100 (pH 7.4) and centrifuged at 10,000 × *g* for 10 min to remove cell debris. A sample volume of 50 μl was mixed with reaction buffer, incubated for 4 h at 37°C, and analyzed by a Spark 10 M multimode microplate reader (Tecan Trading AG).

### Sphingolipid Analysis

For sphingolipid analysis, donor cells were extracted using butanol/methanol (BUME) as previously described (Löfgren et al., 2012). Internal standards [SM 12:0, Cer 17:0, glucosylceramide (GlcCer) 17:0 and lactosylceramide (LacCer) 17:0] were added during the extraction. Prior to analysis, the total extract was exposed to alkaline hydrolysis (0.1 M KOH in methanol) to remove phospholipids that could potentially cause ion suppression effects. The analysis of SM was performed using direct infusion on a QTRAP 5500 mass spectrometer (Sciex) equipped with a robotic nanoflow ion source, the TriVersa NanoMate (Advion BioSciences). Detection was achieved with precursor ion scanning in positive mode using *m/z* 184 (phosphocholine) as a fragment ion (Brugger et al., 1997). The analysis of sphingolipids was performed using UPLC-MS/MS in positive mode, as previously described (Amrutkar et al., 2015). The BioRad BCA kit was used to determine the total cell protein concentration prior to hydrolysis.

### EV Isolation

Conditioned medium from 20 million donor cells was collected 48 h after oα-syn treatment under normoxic or hypoxic conditions. EVs were isolated by differential ultracentrifugation. The conditioned media was spun at 2,000 × *g* for 10 min to remove cells and cell debris, and the supernatant was spun at 10,000 × *g* for 30 min and 100,000 × *g* for 70 min. The EV pellet was washed with PBS and spun again at 100,000 × *g* for 70 min. The final EV pellet was resuspended in 75 μl of PBS for quantification by FluoroCet, 100 μl of PBS for nanoparticle tracking analysis (NTA), or 20 μl of RIPA buffer for immunoblotting and stored at −80°C. The EV size distribution was measured by NTA using the NanoSight NS300 system (Malvern Instruments).

### EV Abundance by FluoroCet

The FluoroCet quantitation kit (SBI) is designed to directly measure acetylcholinesterase (AChE) activity inside of EVs. After EVs were isolated as described above, the assay was performed following the manufacturer’s instructions. The fluorometric assay was read at an excitation/emission of 530/590 nm using a Spark 10M multimode microplate reader.

### Statistical Analysis

The results are presented as the mean ± standard error of the mean (SEM). The statistical significance was analyzed with GraphPad Prism 6 software using Student’s *t* test for all experiments with the exception of lipidomic and RT-PCR analysis, which were completed using one-way ANOVA with Dunnett’s multiple comparison test. Statistical significance was established at *p* ≤ 0.05.

## Results

### Inhibition of nSMase2 and Hypoxia

**FIGURE 2:**
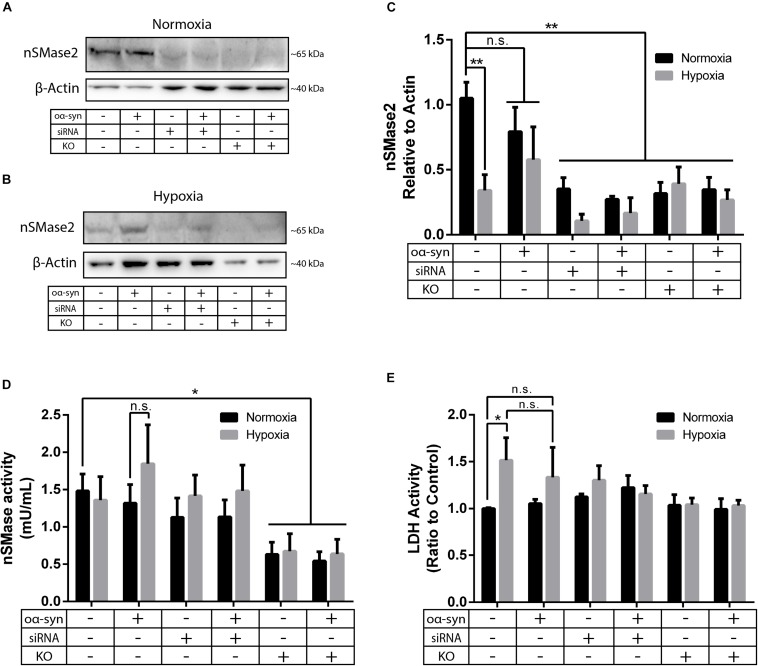
nSMase2 inhibition was achieved by nSMase2 siRNA and KO in donor cells. **(A–C)** Western blot analysis of nSMase2 under **(A)** normoxia and **(B)** hypoxia with **(C)** bar graphs showing its expression relative to β-actin expression (*n* = 3). **(D)** nSMase enzymatic activity was determined by a colorimetric assay kit using cell lysates (*n* = 5). **(E)** Cytotoxicity of nSMase2 KO and siRNA in cells treated with α-syn under normoxia or hypoxia, as determined by LDH activity (*n* = 6). The data are presented as the mean ± SEM. ^∗^*p* ≤ 0.05, ^∗∗^*p* ≤ 0.01.

**FIGURE 3:**
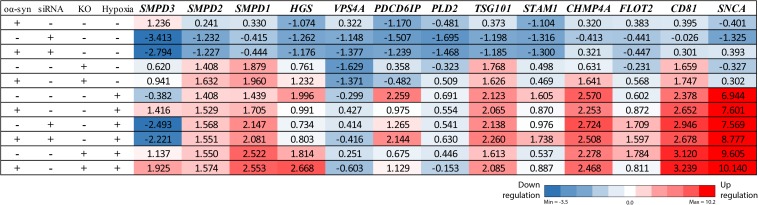
nSMase2 siRNA downregulates the *SMPD3* gene, and ESCRT-dependent genes are upregulated under hypoxia. RT-PCR analysis of common ESCRT genes and *SNCA* (α-syn) in donor cells. Changes in the expression levels are displayed from blue (downregulated) to red (upregulated), with the values representing the mean fold change relative to control samples (*n* = 3). The complete RT-PCR data are available in Supplementary Table S1.

Parkinson’s disease is a common neurodegenerative disease that is characterized by chronic oxidative stress in the brain (Blesa et al., 2015); however, to our knowledge, there are no previous studies investigating nSMase2 activation during hypoxia in human neurons. As the main model for this study, nSMase2 was knocked down in donor cells by either siRNA transfection in SH-SY5Y cells or by the generation of nSMase2 KO SH-SY5Y cells using CRISPR/Cas9. The loss of nSMase2 protein expression in nSMase2 KO cells was confirmed by immunoblotting and immunocytochemistry with N- and C-terminal specific antibodies (Figures 2A–C and Supplementary Figure S1), while siRNA treatment induced both a significant reduction in protein expression and gene downregulation (Figures 2A–C, 3). nSMase enzymatic activity was significantly decreased in KO cells (0.63 ± 0.16 mU/ml) compared to control cells (1.48 ± 0.23 mU/ml) (Figure 2D). However, siRNA-treated cells (1.13 ± 0.25 mU/ml) were not significantly modified, although it is possible that other nSMases are compensating from the acute transfection and are being detected in the assay. Based on these results, we established two different models of nSMase2 inhibition, specifically siRNA-treated cells with a reduction in total nSMase2 protein expression and KO cells with a reduction in total functional enzymatic protein levels.

As a model of oxidative stress, we exposed donor cells to hypoxic conditions of 1% O_2_ for 48 h. Hypoxia significantly reduced nSMase2 protein expression (Figures 2B,C), but nSMase enzymatic activity was not affected (Figure 2D). Compared to normoxia, hypoxia induced cell toxicity, which was completely mitigated by nSMase2 inhibition (Figure 2E), suggesting that a protective mechanism is in play when nSMase2 is reduced.

### nSMase2 Inhibition Prevents oα-syn Aggregation

**FIGURE 4:**
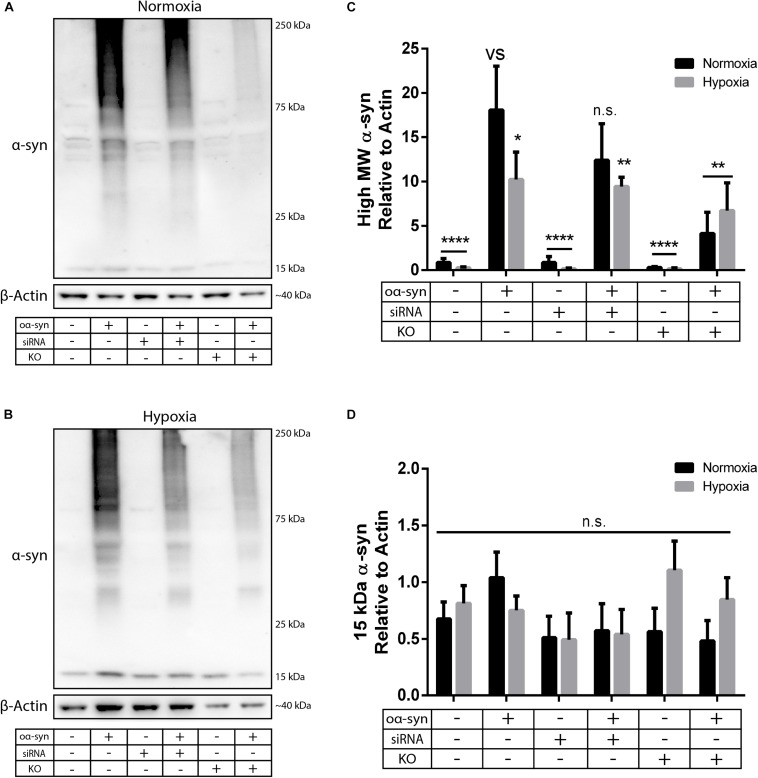
nSMase2 inhibition reduces the aggregation of high molecular weight (MW) α-syn. **(A,B)** Expression of α-syn in donor cells, as determined by Western blot, under **(A)** normoxia and **(B)** hypoxia. **(C,D)** Quantification of **(C)** high MW α-syn (∼25–250 kDa) and **(D)** 15 kDa monomeric α-syn expression relative to β-actin expression. The data are presented as the mean ± SEM (*n* = 3). ^∗^*p* ≤ 0.05, ^∗∗^*p* ≤ 0.01, ^∗∗∗∗^*p* ≤ 0.0001 versus (vs) oα-syn treatment.

Next, we investigated the effect of nSMase2 inhibition on the aggregation of oα-syn, which was modified by the lipid peroxidation product HNE. The elevation of HNE has been observed in the brain during normal aging and is more pronounced in brain areas affected by PD pathology (Yoritaka et al., 1996; Castellani et al., 2002). PTMs caused by HNE contribute to the increased conversion of soluble α-syn monomers into cytotoxic oligomers, as well as increased α-syn secretion from cells (Näsström et al., 2011; Bae et al., 2013). Monomers of α-syn were aggregated in the presence of HNE to produce oα-syn. Size exclusion chromatography was performed to characterize and purify oα-syn, which corresponded to an elution peak with a molecular weight of ∼2000 kDa (Supplementary Figure S2), as described previously (Sardar Sinha et al., 2018). The ultrastructural analysis of the oligomers showed a heterogeneous population of spherical, curvilinear, and ring-like structures (Supplementary Figure S2), as expected (Näsström et al., 2011; Sardar Sinha et al., 2018). Based on previous results from our lab (Domert et al., 2016; Reyes et al., 2019), donor cells were incubated with 1 μM oα-syn for 3 h and analyzed after 48 h. The addition of oα-syn did not contribute to any changes in nSMase2 protein or gene expression under hypoxia or normoxia (Figures 2C, 3). The combination of hypoxia and oα-syn slightly increased nSMase enzymatic activity (1.85 ± 0.52 mU/ml vs. control 1.48 ± 0.23 mU/ml), although this was not statistically significant (Figure 2D; *p* = 0.53). In addition, oα-syn did not increase hypoxia-induced cytotoxicity (Figure 2E). However, the addition of oα-syn resulted in the significant aggregation of high molecular weight (MW) α-syn (25–250 kDa) under both normoxic and hypoxic conditions, an effect that was significantly decreased in KO cells, even under hypoxic conditions (Figures 4A–C). A similar, but non-significant, decrease was also observed in cells subjected to siRNA. Monomeric (∼15 kDa) α-syn levels were not affected by any treatment, despite an increase in SNCA mRNA expression under hypoxia (Figures 3, 4D). Together, these data indicate that nSMase2 may impede the breakdown of aggregated high-molecular-weight α-syn, both under normoxia and hypoxia.

### Hypoxia Promotes Longer Acyl Chain Lengths

**FIGURE 5:**
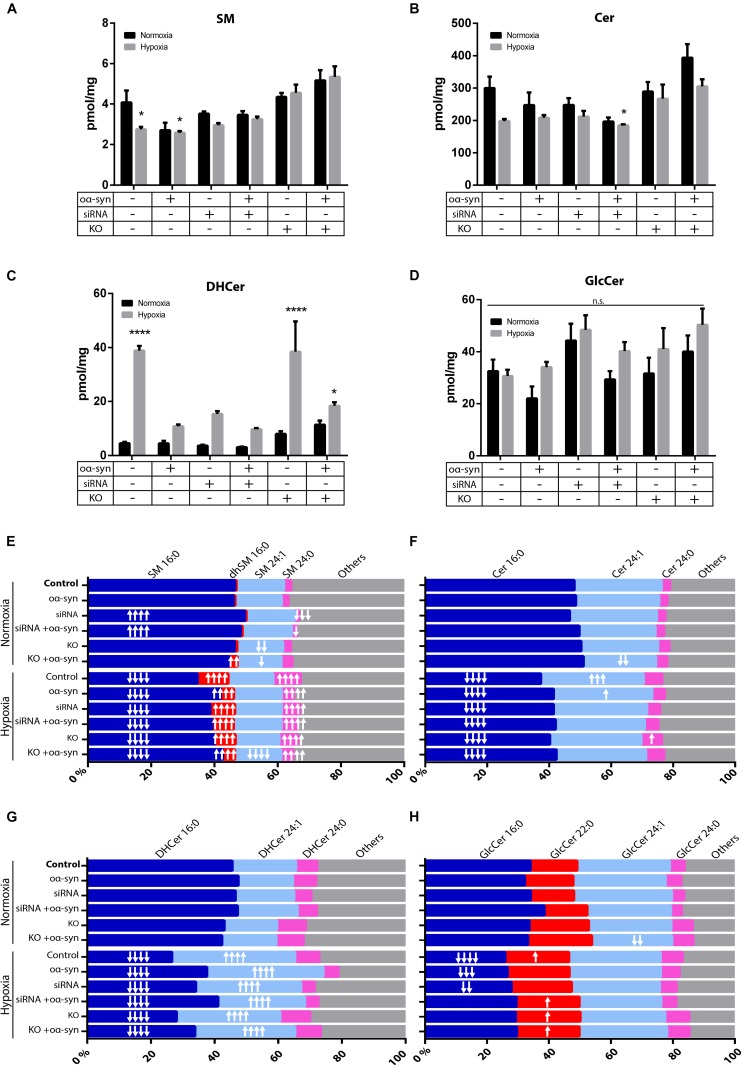
nSMase2 inhibition and oα-syn alleviates acyl chain shift due to hypoxia. **(A–D)** Lipid extraction and UPLC-MS/MS analysis of **(A)** sphingomyelin (SM), **(B)** ceramide (Cer), **(C)** dihydroceramide (DHCer), and **(D)** glucosylceramide (GlcCer) in donor cells. The data are presented as the mean ± SEM (*n* = 4). ^∗^*p* ≤ 0.05, ^∗∗∗∗^*p* ≤ 0.0001 relative to the normoxic control. **(E–H)** For acyl chain length alterations, the data are presented as the mole percentage of **(E)** SM, **(F)** Cer, **(G)** DHCer, and **(H)** GlcCer relative to all acyl chains. Short chains are considered C16–C20, and long chains are considered C21–C24. “↑” denotes an increase and “↓” denotes a decrease, with ↑*p* ≤ 0.05, ↑↑*p* ≤ 0.01, [mymaths]↑↑[mymathe]↑*p* ≤ 0.001, and [mymaths]↑⁣↑⁣↑[mymathe]↑*p* ≤ 0.0001 relative to the normoxic control with respective direction. Detailed lipid information is provided in Supplementary Table S2.

As there have been conflicting results regarding sphingolipids in PD brains (Cheng et al., 2011; Fabelo and Martin, 2011; Abbott et al., 2014; Murphy et al., 2014; Gegg et al., 2015; Bienias et al., 2016), we wanted to investigate whether inhibiting nSMase2 affects the lipid composition of sphingolipids after the addition of oα-syn in the absence or presence of hypoxia using UPLC-MS/MS. Hypoxia alone caused a significant decrease in SM (2.7 ± 0.1 pmol/mg vs. control 4.1 ± 0.6 pmol/mg) and a decrease in Cer, although not significant (198.0 ± 6.8 pmol/mg vs. control 300.1 ± 35.7 pmol/mg; *p* = 0.063) (Figures 5A,B). The inhibition of nSMase2 alone, either by siRNA or KO, did not cause significant differences in total SM or Cer. In siRNA-treated cells, the combined treatment of oα-syn and hypoxia caused a significant reduction in total Cer when compared to the normoxic control (185.0 ± 3.3 pmol/mg vs. control 300 ± 35.7 pmol/mg) (Figure 5B). Cer can also be synthesized by several other lipids, such as dihydroceramide (DHCer), which is converted to Cer through *de novo* synthesis and is associated with slow cell proliferation and cell death [reviewed in Siddique et al. (2015)]. Interestingly, DHCer was significantly increased by hypoxia (38.88 ± 1.77 pmol/mg vs. control 4.50 ± 0.54 pmol/mg), and the effect was mitigated by siRNA or oα-syn treatment but not by KO (Figure 5C). GlcCer, which can also be broken down into Cer, was not significantly dysregulated in any of the treatment groups (Figure 5D).

Cer acyl chain composition has been shown to shift toward shorter acyl chain lengths (C16–C20) in the anterior cingulate cortex in PD patients (Cheng et al., 2011; Abbott et al., 2014), while there is an increase in long-chain SM and Cer (C21-C24) in the visual cortex (Cheng et al., 2011). siRNA treatment resulted in an increased relative percentage of SM 16:0 (49.5 ± 0.6%) and a decreased percentage of SM 24:0 (1.3 ± 0.2%), while nSMase2 KO decreased the percentage of SM 24:1 (14.5 ± 0.1%) relative to that in the control [SM 16:0 (46.4 ± 0.6%), SM 24:0 (2.3 ± 0.1%), and SM 24:1 (15.0 ± 0.5%)] (Figure 5E). oα-syn alone did not significantly change the sphingolipid composition, but nSMase2 KO combined with oα-syn treatment, for example, caused an increase in the percentage of dhSM 16:0 (2.6 ± 0.1%) and Cer 24:1 (23.4 ± 1.8%) compared to those in the control [dhSM 16:0 (0.5 ± 0.1%) and Cer 24:1 (28.1 ± 2.3%)] (Figures 5E,F). Hypoxia alone caused a significant reduction in short acyl chain lengths [SM 16:0 (34.6 ± 0.7%), Cer 16:0 (37.3 ± 1.0%), DHCer 16:0 (26.7 ± 1.0%), and GlcCer 16:0 (25.9 ± 1.7%)] relative to those in the control [SM 16:0 (46.4 ± 0.6%), Cer 16:0 (48.1 ± 2.5%), DHCer 16:0 (45.5 ± 1.3%), and GlcCer 16:0 (34.0 ± 2.8%)] (Figures 5E–H). Consequently, hypoxic conditions alone also promoted longer acyl chain lengths [SM 24:0 (8.7 ± 0.5%), Cer 24:1 (33.1 ± 0.9%), DHCer 24:1 (38.6 ± 1.4%), and GlcCer 22:0 (20.6 ± 1.1%)] relative to those in the control [SM 24:0 (2.3 ± 0.1%), Cer 24:1 (28.1 ± 2.3%), DHCer 24:1 (20.0 ± 0.7%), and GlcCer 22:0 (15.2 ± 0.6%)] (Figures 5E–H). KO and siRNA treatment, as well as oα-syn treatment, contributed to a shift of acyl chain length to shorter chains under hypoxia. Collectively, these data show that nSMase2 inhibition in combination with oα-syn alleviates the lipid shifts that are associated with hypoxic conditions. The complete lipidomic analysis can be found in Supplementary Table S2.

### Transfer of oα-syn Is Reduced by Inhibiting nSMase2

**FIGURE 6:**
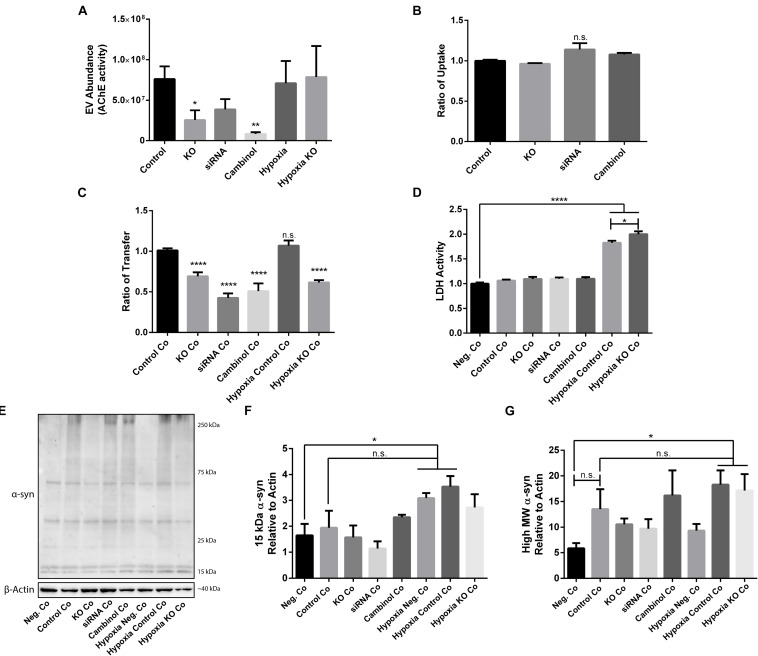
nSMase2 expression is a critical component in the transmission of oα-syn between neuron-like cells. **(A)** EV abundance, as measured by the acetylcholinesterase (AChE) activity, of EVs isolated from donor cells treated with oα-syn (*n* = 4). **(B,C)** Flow cytometry analysis of the oα-syn **(B)** ratio of uptake in donor cells and **(C)** ratio of transfer to recipient cells after 24 h of coculture (Co) (*n* = 6). The provided ratios are relative to WT SH-SY5Y uptake and transfer, where the control = 1.0. **(D)** LDH assay demonstrating cytotoxicity after 24 h of coculture (*n* = 6). **(E–G)** Western blot analysis of **(E)** α-syn after 24 h of coculture and quantifications demonstrating the expression of **(F)** 15-kDa monomeric α-syn and **(G)** high MW α-syn (∼25–250 kDa) relative to that of β-actin. The data are presented as the mean ± SEM relative to the control (*n* = 3). ^∗^*p* ≤ 0.05, ^∗∗^*p* ≤ 0.01, ^∗∗∗∗^*p* ≤ 0.0001.

Due to the importance of nSMase2 in generating EVs through the ESCRT-independent pathway, we next investigated whether the inhibition of nSMase2 affects EV production and its effect on the transmissibility of oα-syn between neuron-like cells. First, we investigated whether oα-syn is found in the EVs. Conditioned media was collected from donor cells 48 h after oα-syn treatment, and EVs were isolated by step-gradient ultracentrifugation. The homogeneity of the EV pellets was confirmed by NTA, which showed a population of enriched vesicles with a mode diameter of 71.61 ± 4.93 nm (Supplementary Figure 3A). Immunoblotting was used to confirm that the EV pellets were enriched with flotillin-1, a common EV marker, and contained oα-syn (Supplementary Figure 3B). For coculture experiments, cambinol, a commercially available nSMase2 inhibitor (Figuera-Losada et al., 2015), was also used to confirm our findings pharmacologically. EV abundance was quantified by directly measuring AChE activity, which is proportional to EV quantity (Savina et al., 2002; Gupta and Knowlton, 2007). After oα-syn treatment, nSMase2 KO, as well as cambinol treatment, resulted in significantly decreased AChE activity, while siRNA treatment reduced AChE activity, although not significantly (*p* = 0.11) (Figure 6A). Hypoxia did not alter the level of AChE activity regardless of whether nSMase2 was knocked out, likely due to the compensation of related ESCRT-related genes (*TSG101*, *CHMP4A*, and *CD81*), which were shown to be upregulated in response to hypoxia (Figure 3).

Since we were able to determine that oα-syn is present in EVs and that nSMase2 reduction also reduced the number of EVs, we aimed to determine if the reduction in EVs also reduces the transfer of oα-syn between neuron-like cells. First, we evaluated whether inhibiting nSMase2 in donor cells impairs oα-syn uptake by analyzing the percentage of cells that take up fluorescently labeled oα-syn using flow cytometry. A reduction in nSMase2 by KO, siRNA, or cambinol did not alter the number of donor cells that readily took up the free oα-syn during the 3 h treatment (Figure 6B). Next, using our well-established donor/recipient coculture model (Agholme et al., 2010; Nath et al., 2012; Domert et al., 2016; Sackmann et al., 2017), oα-syn-loaded donor cells, in which nSMase2 was inhibited, were cocultured onto highly differentiated recipient cells for 24 h and analyzed by flow cytometry. All treatments that inhibited nSMase2 reduced the transfer of oα-syn between donor and recipient cells; KO cells exhibited a reduction of 31 ± 5%, siRNA-treated cells exhibited a reduction of 57 ± 6%, and cambinol-treated cells exhibited a reduction of 50 ± 9% (Figure 6C). Hypoxia did not alter the amount of oα-syn transfer between cells, nor did it influence oα-syn transfer in the nSMase2 KO lineage; the rates of transfer among KO cells and KO cells exposed to hypoxia were not significantly different.

Cocultured cells were vulnerable to hypoxia-induced toxicity (Figure 6D), which caused technical limitations for analyzing donor and recipient cells separately. Hypoxia-exposed cells exhibited an increase in monomeric α-syn compared to that in negative cocultures, but this increase was not significant when compared to control cocultures (Figures 6E,F). A similar effect was observed for high MW α-syn (Figures 6E,G). nSMase2 KO did not provide any protection against hypoxia-induced cytotoxicity in cocultures; in fact, nSMase2 KO cocultures resulted in an increase in cell death compared to hypoxia-exposed cocultures (Figure 6D). nSMase2 KO cocultures displayed a minor reduction in monomeric and high MW α-syn in the presence and absence of hypoxia compared to that in control cocultures, although these differences were not significant (Figures 6E–G). Studies have shown that the phosphorylation of α-syn at serine 129 (pS129) is a disease-specific modification found in Lewy bodies (Fujiwara et al., 2002) and that oxidative stress induces α-syn pS129 (Takahashi et al., 2007). Our model showed a trend toward increased pS129 under hypoxic conditions; however, this increase did not achieve statistical significance (Supplementary Figure S4). Taken together, these data indicate that oα-syn can be readily detected in EVs and that the depletion of the ESCRT-independent related protein nSMase2 causes a substantial reduction in protein propagation between cells. By reducing the production of EVs, we showed that oα-syn transmission was halved even when some EV production persisted. This alludes to the notion that, although other mechanisms of transfer are likely also at play, the cell-to-cell transfer of oα-syn relies heavily upon EVs produced through the ESCRT-independent pathway.

## Discussion

This study is the first to investigate whether the inhibition of nSMase2 reduces the transfer of oα-syn between neuron-like cells. Furthermore, we also examined whether hypoxia contributes to oα-syn propagation in this system. Small EVs, such as exosomes, appear to play an important role in several pathological and physiological processes. EVs mediate the intercellular transfer of cell signaling products, and these particles can carry large aggregated proteins, including oα-syn (Alvarez-Erviti et al., 2011; Danzer et al., 2012; Delenclos et al., 2017). Additionally, exosomal oα-syn has been shown to be taken up more readily than free oα-syn (Danzer et al., 2012), with recent studies showing that exosomal α-syn is found in the CSF (Stuendl et al., 2016) and plasma (Shi et al., 2014) of PD patients, further emphasizing the importance of these vesicles in the pathogenic propagation of α-syn. EVs can be formed through different pathways; however, it is still unclear if these pathways produce distinct populations of EVs and if they depend on context-specific regulation [reviewed in Juan and Fürthauer (2018)]. One of the major pathways by which EVs are created involves the phosphodiesterase enzyme nSMase2, which hydrolyses SM to produce Cer and forms EVs through the ESCRT-independent pathway (Marsh and van Meer, 2008). Several studies have investigated SM metabolism in Alzheimer’s disease [reviewed in Bienias et al. (2016)], and one of these studies found that inhibiting nSMase2 in APP/PS1 mice leads to a reduction in the number of EVs in the brain and serum, a reduction in amyloid-β-42 concentration in plaques, and decreased levels of most Cer species in the serum (Dinkins et al., 2014). Furthermore, it has been shown that inhibiting nSMase2 prevents prion packaging by blocking EV formation (Guo et al., 2015), which is of particular interest given the evidence that misfolded α-syn propagates in a prion-like manner (Luk et al., 2012). Until now, there has been a lack of knowledge in this area regarding PD. Our results showed that inhibiting nSMase2 reduces the amount of high MW α-syn, indicating that nSMase2 is involved in promoting the fibrilization of α-syn or preventing its clearance. Furthermore, inhibiting nSMase2 significantly reduced the amount of oα-syn that was transferred to recipient cells by 31–57%. This effect depends on the transfer mechanisms of the donor cell, not a failure to internalize exogenous oα-syn, as nSMase2 inhibition did not affect the uptake of oα-syn.

Oxidative stress is thought to be an underlying mechanism that leads to cellular dysfunction in PD patients [reviewed in Hwang (2013)], and it has been shown that oxidative stress can activate nSMase2 production and induce apoptosis (Levy et al., 2006; Castillo et al., 2007). However, it is unclear if oxidative stress activates nSMase2 and Cer production in neuronal cells and if this is altered by the presence of oα-syn. Gu et al. determined that, in astrocytes, but not in neurons, nSMase activity increases following brain ischemia in rodents, which in turn leads to neuronal damage (Gu et al., 2013); however, here we show that nSMase2 is mildly sensitive to hypoxia in neuron-like cells. Contrary to this study, we observed that hypoxia reduces nSMase2 at the protein level in neuron-like cells, but this reduction does not correlate with a reduction in enzymatic activity. Oxidative stress may cause modifications that result in increased enzyme activation, even with lower levels of total protein, as it has been suggested that, under normal conditions, nSMase2 primarily exists as an inactive enzyme, while oxidative stress initiates its movement to the plasma membrane where it can generate Cer (Castillo et al., 2007). A similar effect was also observed in the siRNA-treated cells despite them having unaltered enzymatic activity even with reduced protein levels. Since the half-life of nSMase2 in HAE cells has been reported to be 20 h (Filosto et al., 2012), the remaining protein detected after 48 h may also be sufficient for the required enzymatic demands of cells. Levy et al. showed that oxidative stress increases Cer production, inducing cell death (Levy et al., 2006), while another study showed no changes in Cer in response to oxidative stress (Devlin et al., 2011). Others have reported that decreased Cer may be protective under hypoxia by promoting anti-apoptotic conditions (Epstein et al., 2009). In this study, under hypoxia, we observed a decrease in total Cer levels that correlated with an increase in cell death. DHCer is converted to Cer through *de novo* synthesis, correlated with the induction of autophagy [reviewed in Grösch et al. (2012)], and induced by hypoxia (Devlin et al., 2011; Testai et al., 2014). Accordingly, we observed an increase in DHCer after the induction of hypoxia, but this DHCer profile was mitigated after a reduction in nSMase2 and oα-syn treatment, presumably as a protective function to limit apoptosis, as previously described (Dinkins et al., 2014; Shi et al., 2014; Delenclos et al., 2017; Juan and Fürthauer, 2018). Currently, DHCer levels have only been evaluated in the plasma of PD patients (Guedes et al., 2017), but given our findings that reducing nSMase2 alleviates the hypoxia-induced DHCer increase, a further evaluation of this lipid in PD brains would be valuable. As oxidative stress is prevalent in PD, the pharmaceutical inhibition of nSMase2 might prove beneficial.

Changes in acyl chain lengths may be important because EV lipid rafts are influenced by the raft composition of the originating cell (Skotland et al., 2017) and may affect how α-syn binds to them (Kubo et al., 2015). EVs incorporate the lipid rafts of the originating cell during their generation and are enriched in cholesterol, SM, glycosphingolipids, and phosphatidylserine (Bienias et al., 2016). Lipid rafts in PD patients have reduced total SM levels (Fabelo and Martin, 2011), which is similar to what we observed in whole cells under hypoxia, and therefore likely affect affecting lipid raft composition. Similarly, it has been shown that Cer acyl chain composition shifts toward shorter acyl chain lengths at the expense of longer chain lengths in the anterior cingulate in PD brains (Abbott et al., 2014). However, we observed a significant reduction in the short chain SM 16:0, Cer 16:0, and GlcCer 16:0 and an increase in the long chains SM 24:0, Cer 24:1, DHCer 24:1, and GlcCer 22:0 under hypoxic conditions, while nSMase2 inhibition reduced this shift. In aged WT mice, an increase in Cer and SM long chains and a decrease in DHCer short chains have been observed (Vozella et al., 2017), and Cer long chains are associated with mitochondrial damage and cell death (Law et al., 2018); thus, this is a useful model for studying these changes *in vitro.* However, additional research in PD brains needs to be conducted to further elucidate which sphingolipids are dysregulated, as it has been speculated that lipid dysregulation may depend on the brain region investigated (Cheng et al., 2011; Fabelo and Martin, 2011; Abbott et al., 2014; Murphy et al., 2014; Gegg et al., 2015).

The α-syn peptide has a high affinity for lipid rafts, and the lipid composition of these rafts changes the binding behavior of α-syn, affecting its localization to synapses (Fortin et al., 2004; Kubo et al., 2005, 2015). Additionally, synthetic lipid vesicles prepared from neuroblastoma cells accelerate α-syn aggregation (Marie et al., 2015), suggesting that the lipids in EVs themselves are instrumental to the self-aggregation properties of α-syn. Our findings that oα-syn treatment induced an increase in high MW α-syn (∼25–250 kDa) in donor cells while nSMase2 KO cells displayed a significantly reduced amount of high MW α-syn support and extend the importance of nSMase2, and therefore lipid generation and constitution, for α-syn aggregation. Fussi et al. (2018) showed that inhibiting autophagosomes increases exosomal α-syn secretion, presumably to reduce the intracellular α-syn burden. We speculate that blocking EV formation may eliminate one pathway by which cells reduce their intracellular oα-syn burden and thus may upregulate lysosomal–autophagy pathways in an attempt to degrade the α-syn within the cell. Another speculation is that oα-syn may be packed into larger microvesicles that are not as easily taken up by surrounding cells, as it has been reported that inhibiting nSMase2 increases the secretion of larger vesicles originating from the plasma membrane (Menck et al., 2017). Although oxidative stress has been shown to induce α-syn aggregation (Hashimoto et al., 1999; Scudamore et al., 2018), we observed a minor reduction in α-syn aggregation in response to hypoxia, but only in donor cells alone. We also observed an upregulation in α-syn gene expression (SNCA) in donor cells under hypoxia but observed no changes in monomeric α-syn protein expression, possibly due to a reduction in the overall rate of mRNA translation in cells under hypoxic conditions (Koritzinsky and Wouters, 2007). Interestingly, oα-syn treatment in donor cells also contributed to a shift back toward shorter acyl chain lengths, but only under hypoxia. Long acyl chains are more hydrophobic than shorter chains, which causes slower desorption from the plasma membrane (Fielding, 2007) and a disruption in lipid raft formation (Grösch et al., 2012). α-Syn may cause a reduction in lipid complexity in favor of increased desorption in response to hypoxic stress as a cellular defense mechanism. Guitart et al. (2016) showed that the release of EVs carrying prion proteins results in improved survival of neurons under hypoxic conditions. Although we observed a decrease in oα-syn transfer in KO cells under hypoxia, this decrease did not improve cell survival in this system.

It has been shown that hypoxia induces EV secretion (Michael et al., 2012; Kilic et al., 2018), but the degree of EV secretion varies depending on the cell model and method of hypoxia used (Tomazin, 2014; Ramteke et al., 2015). In the model used for this study, hypoxia upregulated many ESCRT-related genes, such as *TSG101*, *CHMP4A*, and *CD81*, which are some of the main components involved in the formation of EVs through ESCRT-dependent pathways (Van Niel et al., 2006; Baietti et al., 2012). However, the protein expression of *CD81* and *TSG101* has been shown to increase in response to hypoxia without affecting the number of total EVs generated (Ramteke et al., 2015), while *CHMP4A* upregulates hypoxia response elements (Shi et al., 2010) and may lead to a decrease in total EVs (Colombo et al., 2013). Our model displayed no change in EV abundance in response to hypoxia in control cells. This indicates that there is a shift toward different EV generation pathways, such as a greater reliance upon the ESCRT-dependent pathway, under hypoxia. This is particularly evident when nSMase2 is reduced; KO, siRNA, and cambinol all significantly reduced the transfer of oα-syn between cells, whereas under hypoxia, cells did not show a reduction in transfer or any reduction in EV abundance. Importantly, the high degree of oα-syn transfer was abolished even under hypoxia when nSMase2 was knocked out. Another study has also indicated that other nSMase isoforms play a role in EV biogenesis, but that the packing of aggregated proteins into EVs was specifically nSMase2 dependent (Guo et al., 2015). This provides strong evidence for the importance of the formation of EVs by nSMase2 as a critical pathway by which oα-syn is propagated between cells.

Our findings show that oα-syn transfer is significantly decreased when nSMase2 is reduced, providing a new avenue for exploring disease-modifying therapeutics for PD. Since these exosomal pathways have been implicated in the propagation of other neurodegenerative diseases, including Alzheimer’s disease, tauopathies, and prion diseases, this could be a useful approach for generalized neuroprotective therapy. However, the direct mechanism by which this is accomplished requires further investigation. This study supports the need for a deeper investigation of sphingolipid metabolism in PD patients to further elucidate the lipid dysregulation and lipid–protein interactions observed in PD.

## Conclusion

In conclusion, we demonstrated that reducing nSMase2 significantly decreases the transfer of oα-syn between neuron-like cells and reduces α-syn aggregation, even under hypoxic conditions. Inhibiting nSMase2 could be a beneficial strategy for reducing the pathogenesis of PD.

## Data Availability

All datasets generated for this study are included in the manuscript and/or the Supplementary Files.

## Author Contributions

VS designed the experimental approach, performed the experiments, analyzed and interpreted the data, generated the figures, participated in the study design, and drafted the manuscript. MS conceived the hypothesis, participated in the study design, designed the experimental approach, and performed the experiments. CS performed the experiments and contributed to the writing of the manuscript. LC and JB performed some experiments. AA-S designed the experimental approach and interpreted the data. MH conceived the hypothesis, coordinated and led the study, and participated in the study design, data interpretation, and writing of the manuscript. All authors read and approved the manuscript.

## Conflict of Interest Statement

The authors declare that the research was conducted in the absence of any commercial or financial relationships that could be construed as a potential conflict of interest.

## Supplementary Material

FIGURE S1 Confirmation of the nSMase2 KO cell line. **(A,B)** KO was confirmed in the nSMase2 KO D3, D9, D12, and C12 clone lines by **(A)** Western blot analysis with an N-terminal antibody and **(B)** in the nSMase2 KO D3 clone line by immunofluorescence using C- and N-terminal antibodies. From these data, nSMase2 KO D3 was selected for use in further studies. Representative confocal images using DAPI (blue) and anti-nSMase2 conjugated to Alexa Fluor 488 (green) (*n* = 3). The scale bar indicates 100 μm.

FIGURE S2 Confirmation of oligomeric α-syn preparations. **(A)** SEC analysis of α-syn to confirm oligomer specificity. **(B)** TEM image of α-syn depicting spherical, ring-like oligomers. α-Syn peptides were heterogeneous in shape and size and could be categorized into three groups, namely, spherical, curvilinear, and ring-like oligomers, based on their shape. The scale bar indicates 500 nm.

FIGURE S3 Characterization of EVs isolated by step gradient ultracentrifugation. **(A)** Size distribution of EVs isolated from donor cells by NTA presented in a representative graph [mode particle size 71.61 ± 4.93 nm (*n* = 8)]. **(B)** Western blot showing the expression of the common EV marker, flotillin-1, and α-syn in EVs isolated from control cells (control EVs), EVs isolated from cells treated with oα-syn (oα-syn EVs), and cell lysates from control cells.

FIGURE S4 Phosphorylation of α-syn at Ser129 is increased under hypoxia but does not achieve statistical significance. **(A)** Expression of pS129 after 24 h of coculture (Co), as determined by Western blot. **(B)** Quantification of the Western blot results relative to β-actin expression. The data are presented as the mean ± SEM (*n* = 3).

TABLE S1 Complete RT-PCR data presented as the Δ*C*_*t*_ values ± SEM (*n* = 3).

TABLE S2 Sphingolipid analysis of SM, Cer, DHCer, GlcCer, and LacCer, as determined by UPLC-MS/MS, presented as pmol/mg or % mol ± SEM (*n* = 4).

## Abbreviations

α-syn: alpha-synuclein
AChE: acetylcholinesterase
Cer: ceramide
DHCer: dihydroceramide
dhSM: dihydrosphingomyelin
ESCRT: endosomal sorting complexes required for transport
EV: extracellular vesicle
GlcCer: glucosylceramide
HNE: 4-hydroxy-2-non-enal
KO: knockout
LacCer: lactosylceramide
nSMase2: neutral sphingomyelinase 2
oα-syn: oligomeric alpha-synuclein
PD: Parkinson’s disease
siRNA: small interfering RNA
SM: sphingomyelin
