# Supplementary figures and images for: Inhibition of nSMase2 Reduces the Transfer of Oligomeric α-Synuclein Irrespective of Hypoxia
Source: Front Mol Neurosci. 2019 Aug 28;12:200. doi: 10.3389/fnmol.2019.00200 (PMC6724746; doi:10.3389/fnmol.2019.00200)

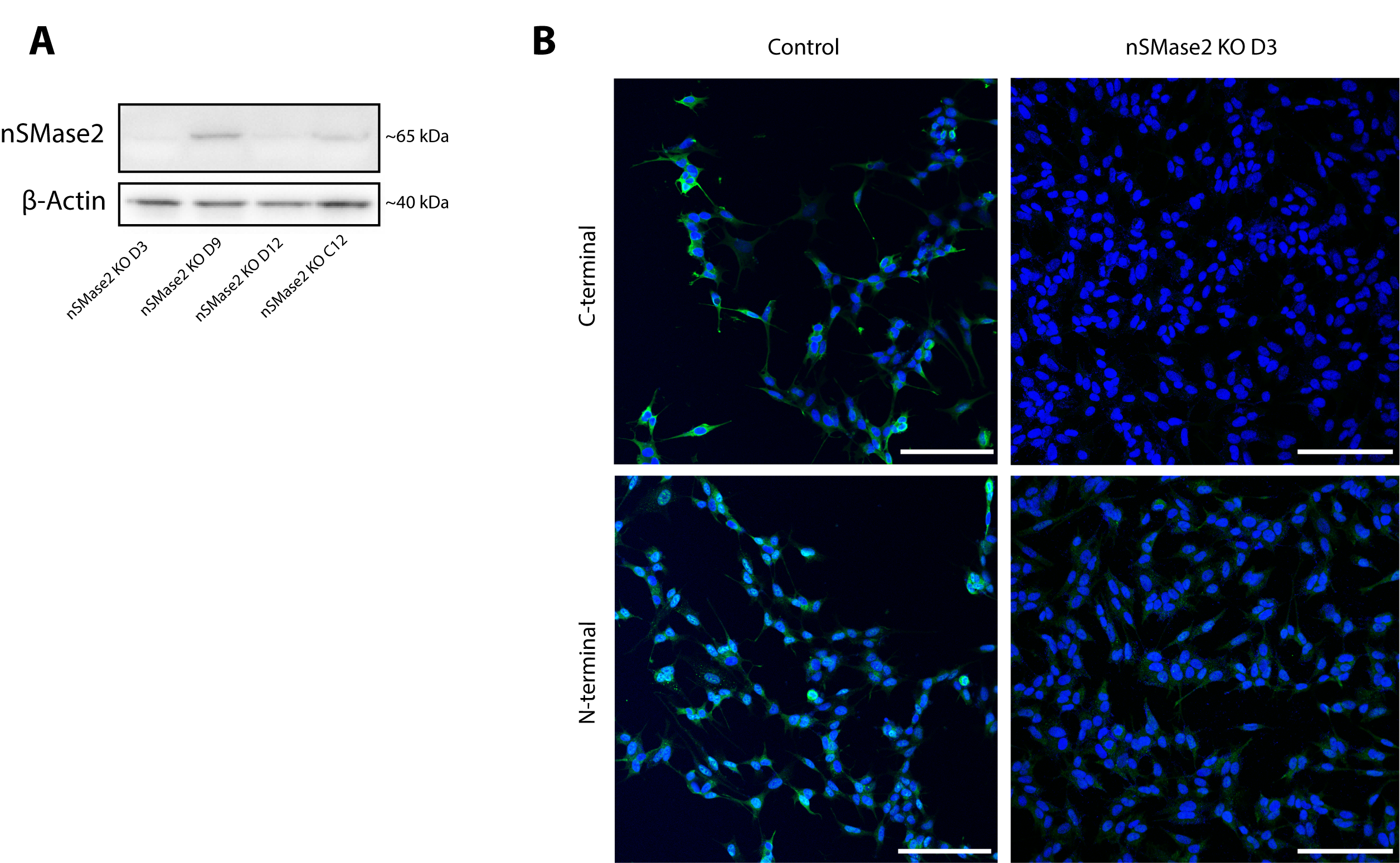

Supplement: FIGURE S1 — Confirmation of the nSMase2 KO cell line. (A,B) KO was confirmed in the nSMase2 KO D3, D9, D12, and C12 clone lines by (A) Western blot analysis with an N-terminal antibody and (B) in the nSMase2 KO D3 clone line by immunofluorescence using C- and N-terminal antibodies. From these data, nSMase2 KO D3 was selected for use in further studies. Representative confocal images using DAPI (blue) and anti-nSMase2 conjugated to Alexa Fluor 488 (green) (n = 3). The scale bar indicates 100 μm. [file Image_1.TIF]

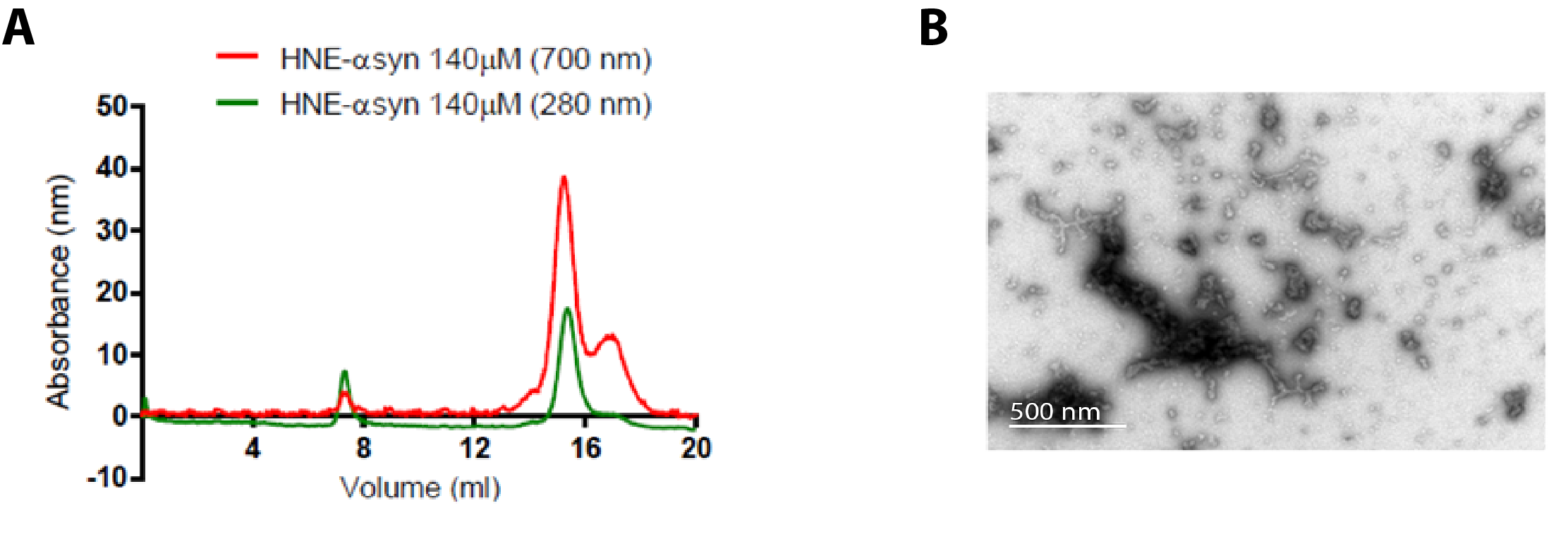

Supplement: FIGURE S2 — Confirmation of oligomeric α-syn preparations. (A) SEC analysis of α-syn to confirm oligomer specificity. (B) TEM image of α-syn depicting spherical, ring-like oligomers. α-Syn peptides were heterogeneous in shape and size and could be categorized into three groups, namely, spherical, curvilinear, and ring-like oligomers, based on their shape. The scale bar indicates 500 nm. [file Image_2.TIF]

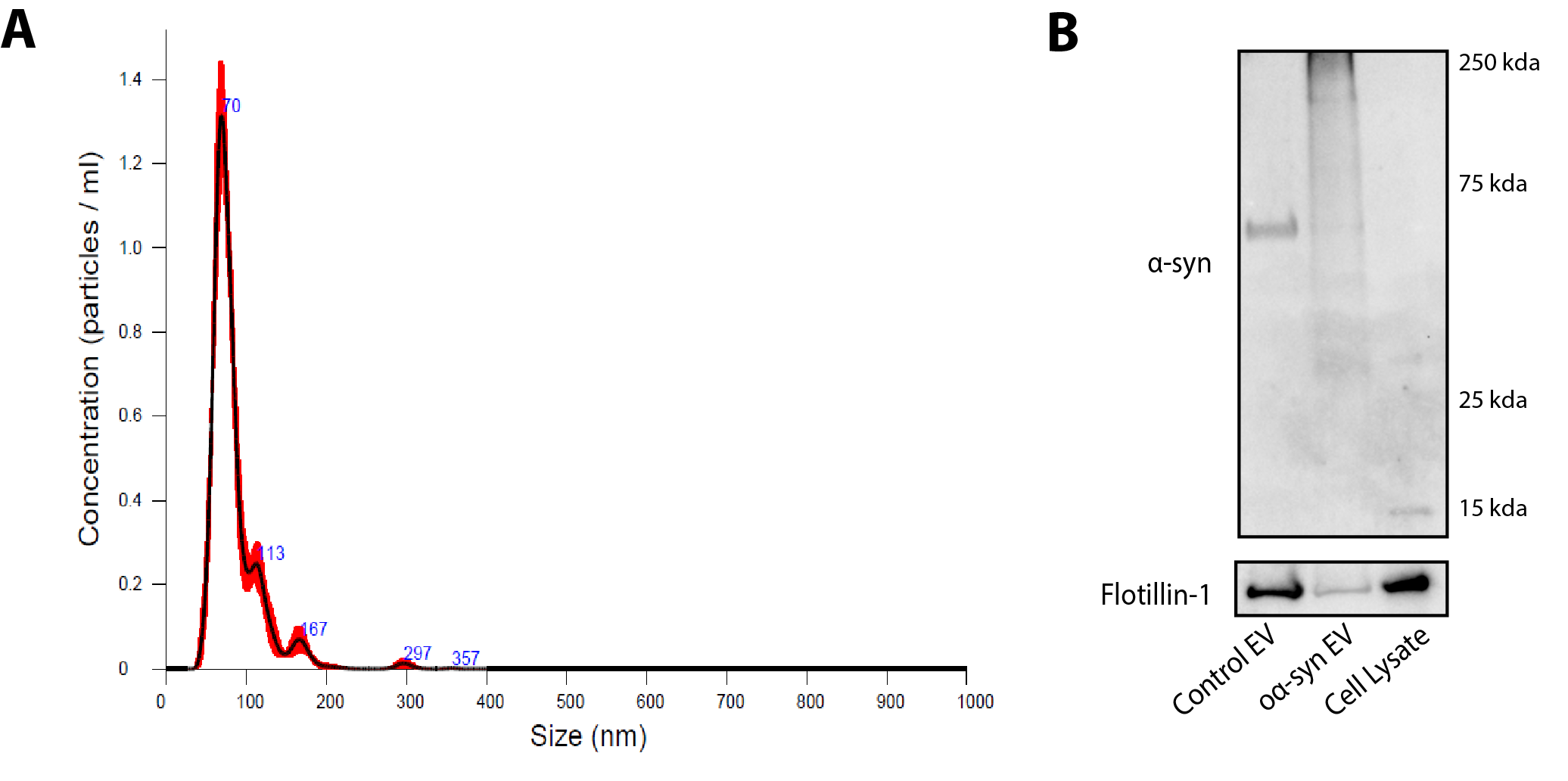

Supplement: FIGURE S3 — Characterization of EVs isolated by step gradient ultracentrifugation. (A) Size distribution of EVs isolated from donor cells by NTA presented in a representative graph [mode particle size 71.61 ± 4.93 nm (n = 8)]. (B) Western blot showing the expression of the common EV marker, flotillin-1, and α-syn in EVs isolated from control cells (control EVs), EVs isolated from cells treated with oα-syn (oα-syn EVs), and cell lysates from control cells. [file Image_3.TIF]

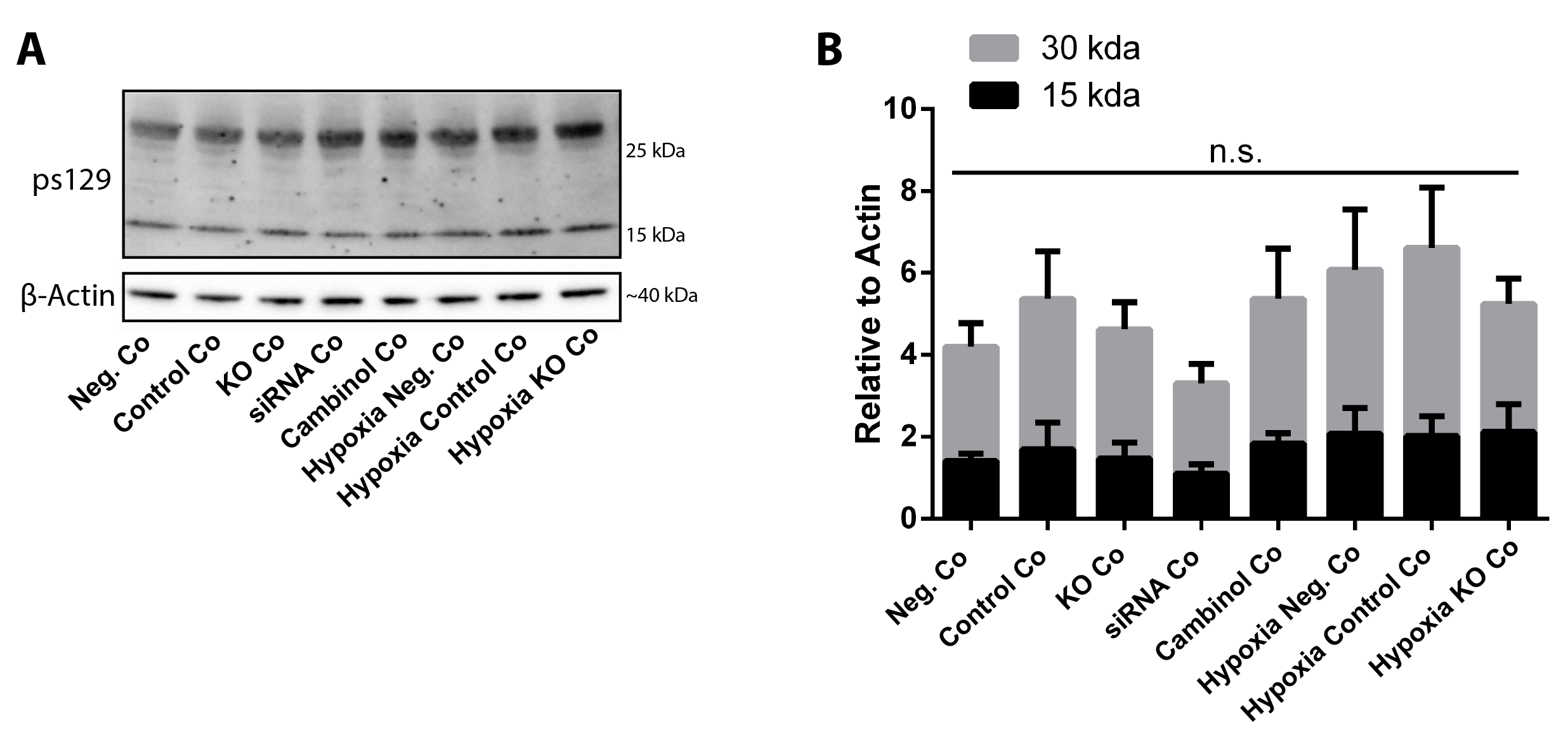

Supplement: FIGURE S4 — Phosphorylation of α-syn at Ser129 is increased under hypoxia but does not achieve statistical significance. (A) Expression of pS129 after 24 h of coculture (Co), as determined by Western blot. (B) Quantification of the Western blot results relative to β-actin expression. The data are presented as the mean ± SEM (n = 3). [file Image_4.TIF]
